# Racial and Ethnic Differences in Amyloid PET Positivity in Individuals With Mild Cognitive Impairment or Dementia: A Secondary Analysis of the Imaging Dementia–Evidence for Amyloid Scanning (IDEAS) Cohort Study

**DOI:** 10.1001/jamaneurol.2022.3157

**Published:** 2022-10-03

**Authors:** Consuelo H. Wilkins, Charles C. Windon, Peggye Dilworth-Anderson, Justin Romanoff, Constantine Gatsonis, Lucy Hanna, Charles Apgar, Ilana F. Gareen, Carl V. Hill, Bruce E. Hillner, Andrew March, Barry A. Siegel, Rachel A. Whitmer, Maria C. Carrillo, Gil D. Rabinovici

**Affiliations:** 1 Department of Medicine, Division of Geriatric Medicine, Vanderbilt University Medical Center, Nashville, Tennessee; 2 Department of Neurology, Memory and Aging Center, Weill Institute for Neurosciences, University of California, San Francisco; 3 Health Policy and Management, Gillings School of Global Public Health, University of North Carolina at Chapel Hill, Chapel Hill; 4 Center for Statistical Sciences, Brown University School of Public Health, Providence, Rhode Island; 5 Department of Epidemiology, Brown University School of Public Health, Providence, Rhode Island; 6 Center for Research and Innovation, American College of Radiology, Reston, Virginia; 7 Alzheimer’s Association, Chicago, Illinois; 8 Department of Medicine, Virginia Commonwealth University, Richmond; 9 Center for Research and Innovation, American College of Radiology, Philadelphia, Pennsylvania; 10 Edward Mallinckrodt Institute of Radiology, Washington University School of Medicine, St Louis, Missouri; 11 Division of Research, Kaiser Permanente, Oakland, California; 12 Department of Public Health Sciences, University of California, Davis; 13 Associate Editor, *JAMA Neurology*; 14 Department of Radiology & Biomedical Imaging, University of California, San Francisco

## Abstract

**Importance:**

Racial and ethnic groups with higher rates of clinical Alzheimer disease (AD) are underrepresented in studies of AD biomarkers, including amyloid positron emission tomography (PET).

**Objective:**

To compare amyloid PET positivity among a diverse cohort of individuals with mild cognitive impairment (MCI) or dementia.

**Design, Setting, and Participants:**

Secondary analysis of the Imaging Dementia–Evidence for Amyloid Scanning (IDEAS), a single-arm multisite cohort study of Medicare beneficiaries who met appropriate-use criteria for amyloid PET imaging between February 2016 and September 2017 with follow-up through January 2018. Data were analyzed between April 2020 and January 2022. This study used 2 approaches: the McNemar test to compare amyloid PET positivity proportions between matched racial and ethnic groups and multivariable logistic regression to assess the odds of having a positive amyloid PET scan. IDEAS enrolled participants at 595 US dementia specialist practices. A total of 21 949 were enrolled and 4842 (22%) were excluded from the present analysis due to protocol violations, not receiving an amyloid PET scan, not having a positive or negative scan, or because of small numbers in some subgroups.

**Exposures:**

In the IDEAS study, participants underwent a single amyloid PET scan.

**Main Outcomes and Measures:**

The main outcomes were amyloid PET positivity proportions and odds.

**Results:**

Data from 17 107 individuals (321 Asian, 635 Black, 829 Hispanic, and 15 322 White) with MCI or dementia and amyloid PET were analyzed between April 2020 and January 2022. The median (range) age of participants was 75 (65-105) years; 8769 participants (51.3%) were female and 8338 (48.7%) were male. In the optimal 1:1 matching analysis (n = 3154), White participants had a greater proportion of positive amyloid PET scans compared with Asian participants (181 of 313; 57.8%; 95% CI, 52.3-63.2 vs 142 of 313; 45.4%; 95% CI, 39.9-50.9, respectively; *P* = .001) and Hispanic participants (482 of 780; 61.8%; 95% CI, 58.3-65.1 vs 425 of 780; 54.5%; 95% CI, 51.0-58.0, respectively; *P* = .003) but not Black participants (359 of 615; 58.4%; 95% CI, 54.4-62.2 vs 333 of 615; 54.1%; 95% CI, 50.2-58.0, respectively; *P* = .13). In the adjusted model, the odds of having a positive amyloid PET scan were lower for Asian participants (odds ratio [OR], 0.47; 95% CI, 0.37-0.59; *P* < .001), Black participants (OR, 0.71; 95% CI, 0.60-0.84; *P* < .001), and Hispanic participants (OR, 0.68; 95% CI, 0.59-0.79; *P* < .001) compared with White participants.

**Conclusions and Relevance:**

Racial and ethnic differences found in amyloid PET positivity among individuals with MCI and dementia in this study may indicate differences in underlying etiology of cognitive impairment and guide future treatment and prevention approaches.

## Key Points

**Question:**

Do amyloid positron emission tomography (PET) positivity rates differ across racial and ethnic groups with mild cognitive impairment (MCI) or dementia?

**Findings:**

In this cohort study of 17 107 Medicare beneficiaries with MCI or dementia, the proportion of amyloid positive PET scans was greater among White participants compared with Black and Asian participants. When racial and ethnic groups were matched by social and demographic factors, the proportion of amyloid positive PET scans was greater among White participants compared with Hispanic and Asian participants but not compared with Black participants.

**Meaning:**

The results of this study showed differences in rates of amyloid PET positivity among racial and ethnic groups; these findings may reflect differences in underlying etiology of cognitive impairment between groups.

## Teaser

This cohort study evaluates differences by race and ethnicity in participants with mild cognitive impairment or dementia.

## Introduction

The prevalence of Alzheimer disease (AD) is rapidly increasing in the aging population and is projected to nearly triple in the coming decades.^1^ Black and Hispanic individuals are 1.5 to 2 times more likely to be diagnosed with clinical AD or related dementias (ADRD) compared with other racial and ethnic groups.^2,3^ In contrast, Asian American individuals (across subgroups) in the US may have lower age-adjusted incidence of all-cause dementia.^4^ The increased risk of ADRD among Black and Hispanic individuals may be driven by dementia risk factors, including rates of cardiovascular disease and diabetes,^5^ as well as social and structural factors (eg, lived experiences of discrimination and racism, economic opportunity, neighborhood disadvantage, and access to quality education).^6,7,8^ Disparities in ADRD are further exacerbated by delayed diagnosis and misdiagnosis of AD,^9^ lack of access to dementia specialist practices,^9^ and biases in neuropsychological testing^10,11^ impacting minoritized racial groups.

The emergence of novel molecular therapies for AD (eg, the recently approved anti-amyloid monoclonal antibody aducanumab) highlight the importance of diagnosis at an early clinical stage and biomarker confirmation of AD pathology among patients who are potential candidates for disease-modifying therapy.^12^ Pathologically, AD is characterized by β-amyloid and τ deposition in the brain with amyloid plaques representing a core feature of disease.^13^ Presently, there are 3 positron emission tomography (PET) radiopharmaceuticals approved by the US Food and Drug Administration (FDA), namely fluorine 18 (^18^F)–labeled florbetapir, ^18^F-labeled flutemetamol, and ^18^F-labeled florbetaben, for the in-vivo detection of amyloid plaques. Unfortunately, amyloid PET biomarkers have largely been studied in individuals identified as White, with minimal inclusion of racially and ethnically diverse groups.^14,15,16,17,18^ Studies examining racial and ethnic differences in amyloid PET have yielded variable results and have primarily included cognitively unimpaired participants.^18,19,20,21,22^ Given the importance of biomarkers like amyloid PET in the early and accurate diagnosis of AD, understanding racial and ethnic differences in amyloid PET positivity is crucial for improving the management of ADRD among diverse groups.

The Imaging Dementia–Evidence for Amyloid Scanning (IDEAS)^23^ study assessed the utility of amyloid PET in Medicare beneficiaries across the US with MCI or dementia in a large national network of dementia specialists. In the present secondary data analyses, we compare amyloid PET results across racial and ethnic groups from IDEAS and compare sociodemographic and comorbidity data to further explore racial and ethnic differences in ADRD clinical presentations and risk factors.

## Methods

IDEAS^23^ examined the association between β-amyloid PET and changes in patient management and patient-oriented outcomes in Medicare beneficiaries with MCI or dementia. The practice-based, pragmatic study engaged 946 dementia specialists from 595 practices who recruited and referred Medicare beneficiaries for amyloid PET imaging at 343 imaging facilities across the US. Study design and report on the associations between amyloid PET and changes in patient management have been previously published, along with the full protocol and study materials.^23^

### Study Design

#### Population

The population in IDEAS consisted of Medicare beneficiaries 65 years and older diagnosed with MCI or dementia by a dementia specialist and in whom the cause of cognitive impairment was uncertain after a comprehensive clinical evaluation and knowledge of amyloid status was expected to impact diagnosis and management.^23^ Of the 21 949 participants enrolled in IDEAS, 18 256 (83.2%) were considered for inclusion in this study. Exclusions included protocol violations (n = 319), not receiving an amyloid PET scan (n = 3337), and not having a positive or negative scan result (n = 37). Of 18 256 participants considered for inclusion, participants who were identified as multiracial (n = 31), Indigenous (n = 32), or had unknown or unreported race or ethnicity (n = 1086) were excluded owing to small numbers, resulting in 17 107 total participants available for analyses (Figure).

**Figure.**
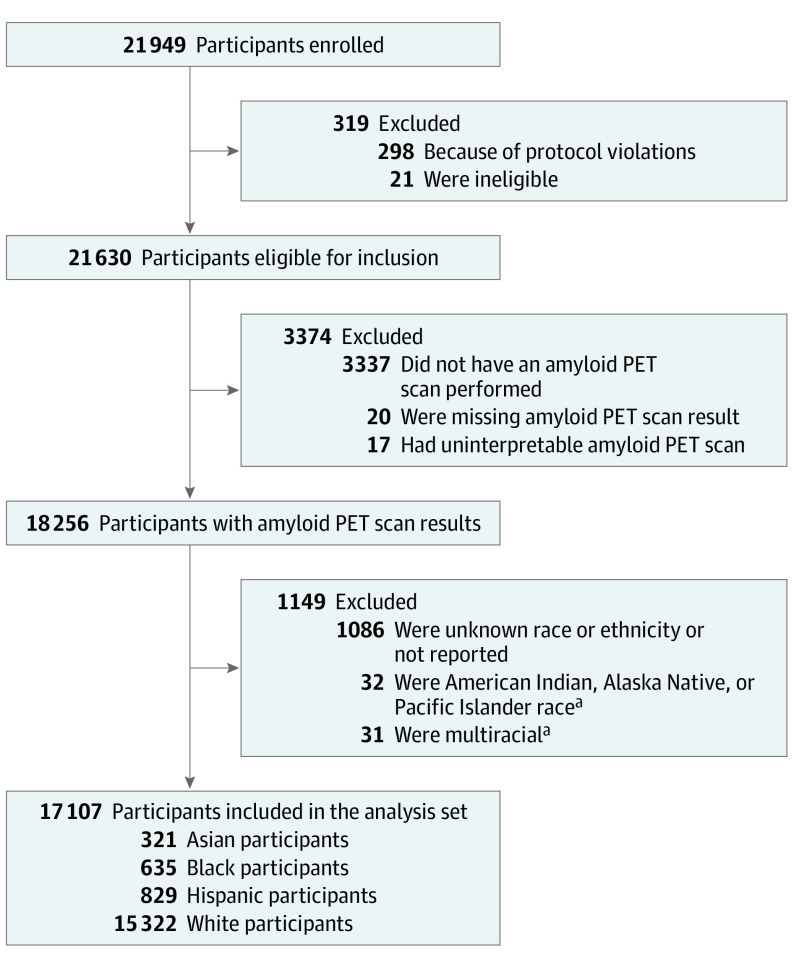
Flow Diagram of Participant Selection PET indicates positron emission tomography. ^a^Participants with American Indian, Alaska Native, and Pacific Islander race and multiracial participants were excluded because of small sample size.

Race and ethnicity in IDEAS were recorded during study registration by dementia specialists, and it is unknown whether race and ethnicity were ascertained by patient report (eMethods in the Supplement). Options for race included American Indian, Alaska Native, Asian, Black or African American, Native Hawaiian or Pacific Islander, White, not reported, or unknown. More than 1 race could be selected. Ethnicity was recorded as Hispanic or Latino, not Hispanic or Latino, not reported, or unknown.

A single variable was created to summarize participant race and ethnicity for the purposes of analysis in the present study (eResults in the Supplement). Individuals identified as Hispanic or Latino ethnicity, regardless of race, were categorized as Hispanic. Individuals indicating multiple races were categorized as multiracial and were not included in the comparisons. Individuals identified as Indigenous (American Indian, Native American, Alaska Native, Native Hawaiian, and Pacific Islander) were not included in the comparison. All other individuals were categorized by the 1 race they selected: Asian, Black, or White.

#### Informed Consent in IDEAS

The IDEAS study used a central institutional review board (Advarra, formerly Schulman Associates) and was managed by the American College of Radiology. Participants provided written consent to allow for their data to be used for future research purposes. PET results are reported according to standards for studies of diagnostic test accuracy in dementia.^24^

### Statistical Analyses

Baseline participant and disease characteristics were summarized by median and range for continuous variables or by counts and percentages for categorical variables for each coded racial or ethnic group (eResults in the Supplement). The proportion of participants with a positive amyloid PET scan result and corresponding 95% Wilson confidence interval (CI)^25^ were calculated for each racial and ethnic group as well as in subsets defined by impairment level.

Because of differences in social factors (eg, educational attainment) and medical history across racial and ethnic groups, we performed optimal 1:1 matching to compare amyloid PET positivity between racial and ethnic minority groups and White participants (eMethods in the Supplement). Variables for matching^26^ were selected based on their association with the outcome of interest (amyloid PET positivity) and included age (matching within ±3 years), sex, highest level of education attained, living arrangement (with whom do you reside; coded into living alone vs not living alone for purposes of analyses), history of hypertension, history of diabetes, family history of dementia, and level of impairment (MCI or dementia). For each racial and ethnic group, the proportion of amyloid-positive participants was calculated along with its corresponding 95% Wilson CI.^25^ The amyloid PET scan results for each racial and ethnic minority group were compared to the amyloid PET scan results of White participants using the McNemar test.

Because matching reduced the sample by 82% (from 17 107 to 3154), we were concerned the matching analysis was not powered to detect significant changes. Thus using all data available for analyses (the full analysis set), we also conducted a multivariable logistic regression model where age, sex, highest level of education attained, living arrangement, history of hypertension, history of diabetes, family history of dementia, level of impairment (MCI vs dementia), and race and ethnicity were added as covariates and amyloid PET scan result was used as the outcome. The association between each covariate and amyloid PET scan result was summarized by odds ratios (ORs) and 95% Wald CIs. Wald tests evaluating whether the odds of having a positive scan were equal were also performed. All reported *P* values are from 2-tailed tests at a significance level of .05. These analyses were exploratory in nature and were not adjusted for multiplicity. All statistical analyses were performed using SAS/STAT version 9.4 (SAS Institute).

## Results

A total of 17 107 participants were included in the full analysis set, including 321 Asian individuals (1.9%), 635 Black individuals (3.7%), 829 Hispanic individuals (4.8%), and 15 322 White (89.6%) individuals. The median (range) age of participants was 75 (65-105) years; 8769 participants (51.3%) were female and 8338 (48.7%) were male. Additional sociodemographic characteristics and information about health comorbidities are listed in Table 1.

**Table 1.** Participant Demographic Characteristics

Variable	Race and ethnicity, No. (%)				
	Total	Asian	Black	Hispanic	White
No.	17 107	321	635	829	15 322
Age, median (range), y	75 (65-105)	76 (65-93)	75 (65-95)	76 (65-96)	75 (65-105)
Sex					
Female	8769 (51.3)	171 (53.3)	399 (62.8)	515 (62.1)	7684 (50.2)
Male	8338 (48.7)	150 (46.7)	236 (37.2)	314 (37.9)	7638 (49.8)
Education					
<High school	1160 (6.8)	41 (12.8)	110 (17.3)	316 (38.1)	693 (4.5)
High school (including equivalency)	4527 (26.5)	62 (19.3)	217 (34.2)	205 (24.7)	4043 (26.4)
Some college or associate’s degree	4001 (23.4)	40 (12.5)	131 (20.6)	150 (18.1)	3680 (24.0)
Bachelor’s degree	3994 (23.3)	99 (30.8)	89 (14.0)	74 (8.9)	3732 (24.4)
Master’s degree	2032 (11.9)	31 (9.7)	58 (9.1)	44 (5.3)	1899 (12.4)
Doctorate	1393 (8.1)	48 (15.0)	30 (4.7)	40 (4.8)	1275 (8.3)
Living arrangement					
Patient lives alone	3057 (17.9)	36 (11.2)	160 (25.2)	130 (15.7)	2731 (17.8)
Patient lives with ≥ 1other person	14 050 (82.1)	285 (88.8)	475 (74.8)	699 (84.3)	12 591 (82.2)
History of hypertension					
No	8386 (49.0)	171 (53.3)	203 (32.0)	380 (45.8)	7632 (49.8)
Yes	8721 (51.0)	150 (46.7)	432 (68.0)	449 (54.2)	7690 (50.2)
History of diabetes					
No	14 352 (83.9)	241 (75.1)	458 (72.1)	608 (73.3)	13 045 (85.1)
Yes	2755 (16.1)	80 (24.9)	177 (27.9)	221 (26.7)	2277 (14.9)
Family history of dementia					
No	12 907 (75.4)	279 (86.9)	531 (83.6)	682 (82.3)	11 415 (74.5)
Yes	4200 (24.6)	42 (13.1)	104 (16.4)	147 (17.7)	3907 (25.5)
Impairment level					
MCI	10 400 (60.8)	169 (52.6)	305 (48.0)	370 (44.6)	9556 (62.4)
Dementia	6707 (39.2)	152 (47.4)	330 (52.0)	459 (55.4)	5766 (37.6)

Abbreviation: MCI, mild cognitive impairment.

Results of the 1:1 matching, which included 3154 participants (313 Asian to 313 White, 615 Black to 615 White, 780 Hispanic to 780 White), matched by age, sex, educational attainment, living arrangement, personal history of hypertension, personal history of diabetes, family history of dementia, and level of cognitive impairment, are included in the eResults in the Supplement. We were unable to identify matches for 8 Asian participants (2.5%), 20 Black participants (3.1%), and 49 Hispanic participants (5.9%). After matching, White participants were more likely to have a positive amyloid PET compared with Asian participants (181 of 313; 57.8%; 95% CI, 52.3-63.2 vs 142 of 313; 45.4%; 95% CI, 39.9-50.9, respectively; *P* = .001) and Hispanic participants (482 of 780; 61.8%; 95% CI, 58.3-65.1 vs 425 of 780; 54.5%; 95% CI, 51.0-58.0, respectively; *P* = .003) but not Black participants (359 of 615; 58.4%; 95% CI, 54.4-62.2 vs 333 of 615; 54.1%; 95% CI, 50.2-58.0, respectively; *P* = .13) (Table 2). When examined within strata of level of impairment (MCI or dementia), amyloid positivity differences between Hispanic participants and White participants with MCI were no longer significant (190 of 356 [53.4%] vs 164 of 356 [46.1%] respectively; *P* = .05) but remained significant for those with dementia (292 of 424 [68.9%] vs 261 of 424 [61.6%], respectively; *P* = .02). Conversely, amyloid positivity differences between Asian participants and White participants with dementia were no longer significant (92 of 145 [63.4%] vs 81 of 145 [55.9%], respectively; *P* = .18) but remained significant for those with MCI (89 of 168 [53.0%] vs 61 of 168 [36.3%], respectively; *P* = .002). There were no significant amyloid positivity differences between matched Black or African American and White participants when stratified by level of impairment: 128 of 302 (42.4%) vs 149 of 302 (49.3%), respectively (*P* = .10) and 205 of 313 (65.5%) vs 210 of 313 (67.1%), respectively (*P* = .65) for those with dementia (Table 2).

**Table 2.** Amyloid Positivity Differences Between 1:1 Matched Participants

Amyloid PET scan result	Matched participants, No. (%)					
	Asian	White	Black	White	Hispanic	White
No.	313	313	615	615	780	780
**MCI and dementia**						
Positive, No. (%) [95% CI]	142 (45.4) [39.9-50.9]	181 (57.8) [52.3-63.2]	333 (54.1) [50.2-58.0]	359 (58.4) [54.4-62.2]	425 (54.5) [51.0-58.0]	482 (61.8) [58.3-65.1]
Negative	171 (54.6)	132 (42.2)	282 (45.9)	256 (41.6)	355 (45.5)	298 (38.2)
**MCI**						
Positive	61 (36.3)	89 (53.0)	128 (42.4)	149 (49.3)	164 (46.1)	190 (53.4)
Negative	107 (63.7)	79 (47.0)	174 (57.6)	153 (50.7)	192 (53.9)	166 (46.6)
**Dementia**						
Positive	81 (55.9)	92 (63.4)	205 (65.5)	210 (67.1)	261 (61.6)	292 (68.9)
Negative	64 (44.1)	53 (36.6)	108 (34.5)	103 (32.9)	163 (38.4)	132 (31.1)

Abbreviations: MCI, mild cognitive impairment; PET, positron emission tomography.

The results of the logistic regression model including all participants in the full analysis set (N = 17 107) and adjusting for all matching variables are shown in Table 3. In this adjusted model, the odds of having a positive amyloid PET scan were significantly lower for Asian participants (OR, 0.47; 95%, CI 0.37-0.59; *P* < .001) and Black participants (OR, 0.71; 95% CI, 0.60-0.84; *P* < .001), and Hispanic participants (OR, 0.68; 95% CI, 0.59-0.79; *P* < .001) compared with White participants. Increasing age (OR, 1.36; 95% CI, 1.30-1.44; *P* < .001), female sex (OR, 1.20; 95% CI, 1.12-1.28; *P* < .001), bachelor’s degree educational attainment (OR, 1.24; 95% CI, 1.07-1.44; *P* = .004), master’s degree educational attainment (OR, 1.24; 95% CI, 1.06-1.45; *P* = .009), living with another person (OR, 1.19; 95% CI, 1.09-1.29; *P* < .001), and family history of dementia (OR, 1.36; 95% CI, 1.27-1.47; *P* < .001) were all associated with increased odds of having a positive amyloid PET scan (Table 3).

**Table 3.** Multivariable Logistic Regression Model Adjusting for All Matching Variables (N = 17 107)

Variable	OR (95% CI)	*P* value^a^
Intercept	0.09 (0.06-0.14)	
Age (10 y)	1.36 (1.30-1.44)	<.001
Sex		
Female	1.20 (1.12-1.28)	<.001
Male	1 [Reference]	NA
Education		
Doctorate	1.11 (0.93-1.32)	.24
Master’s degree	1.24 (1.06-1.45)	.009
Bachelor’s degree	1.24 (1.07-1.44)	.004
Some college or associate’s degree	1.05 (0.91-1.21)	.51
High school (including equivalency)	1.12 (0.97-1.29)	.11
<High school	1 [Reference]	NA
Living arrangement		
Patient lives with at least 1 other person	1.19 (1.09-1.29)	<.001
Patient lives alone	1 [Reference]	NA
History of hypertension		
Yes	0.94 (0.88-1.01)	.08
No	1 [Reference]	NA
History of diabetes		
Yes	0.68 (0.62-0.74)	<.001
No	1 [Reference]	NA
Family history of dementia		
Yes	1.36 (1.27-1.47)	<.001
No	1 [Reference]	NA
Impairment level		
Dementia	1.93 (1.81-2.07)	<.001
MCI	1 [Reference]	NA
Race and ethnicity		
Asian	0.47 (0.37-0.59)	<.001
Black	0.71 (0.60-0.84)	<.001
Hispanic	0.68 (0.59-0.79)	<.001
White	1 [Reference]	NA

Abbreviations: MCI, mild cognitive impairment; NA, not applicable; OR, odds ratio.

^a^ *P* values are from the Wald test.

## Discussion

Our study of a large, national cohort of community-dwelling Medicare beneficiaries with MCI or dementia revealed lower proportions and odds of amyloid PET positivity among minoritized racial and ethnic groups. The proportion of amyloid PET positivity among Asian individuals and Hispanic individuals with MCI and dementia was 7% to 12% lower than matched White individuals and 4% lower among Black participants, although these differences were not statistically significant. Asian, Black, and Hispanic participants had lower odds (0.47-0.71) of amyloid PET positivity than White participants. With 1785 individuals who were Asian, Black, or Hispanic, this study includes one of the largest samples of individuals with cognitive impairment from minoritized racial and ethnic groups receiving amyloid PET imaging.^27,28,29^

Despite disproportionately higher rates of dementia and clinical AD among Black and Hispanic populations, we found lower odds of amyloid PET positivity among Black and Hispanic participants compared with White participants. These findings may reflect differences in the etiology of cognitive impairment, such as underlying vascular disease or social factors that are impacting health. Black^30,31,32,33^ and Hispanic individuals^30,34^ have higher age-adjusted rates of hypertension^35,36,37,38,39^ and diabetes,^40,41^ which are associated with increased white matter pathology, cortical and lacunar infarcts, microinfarcts, and nonamyloid and nonvascular pathology.

There are implications of our findings in the context of the recently approved disease-modifying anti-amyloid monoclonal antibody aducanumab. This drug received FDA approval for treatment of MCI or mild dementia due to AD based on lowering of amyloid PET signal as a surrogate biomarker. Lack of inclusivity of diverse populations in clinical trials for aducanumab (2 phase 3 studies included 10% Asian participants, 1% Black, and 3.4% Hispanic) along with our findings must be considered in the context of recommendations for novel therapies.^17^ If diverse groups are less likely to benefit from amyloid-directed therapies and likely to experience considerable financial hardship from the associated cost, there is a risk these novel treatment options may exacerbate existing racial and ethnic disparities in dementia care. Additionally, we found more Black and Hispanic participants with dementia (vs MCI) in our study, which may have been the result of referral bias in the IDEAS study, but may also be representative of disparities in level of impairment at presentation. This has important therapy implications given those with more advanced impairment may not be eligible for novel therapies.

Our findings are the result of 2 different analytic approaches, which were chosen to address group differences in sociodemographic factors and medical history (1:1 matching) and underrepresentation of minoritized racial and ethnic groups in the study (multivariable logistic regression). Because there are known differences (ie, educational attainment, hypertension, and diabetes) and likely unknown or uncaptured factors (such as neighborhood disadvantage and structural racism) associated with the racialization of groups, we chose to match individuals based on variables collected that could potentially impact cognitive status and amyloid PET results. However, matching reduced our sample size by 82% (from 17 107 to 3154) leading to concerns that the matched sample size would be insufficient to detect differences. Hence we also performed a multivariable logistic regression to use all available data. Although sociodemographic and comorbidity factors are interconnected with race and ethnicity as a result of structural racism and systemic inequality within the US,^42,43^ these were treated as independent variables in the logistic regression model.

Although we found lower proportions and odds of amyloid PET positivity for Asian and Hispanic participants, Black participants had significantly lower odds but no difference in proportion. As noted above, this may reflect insufficient sample size after matching to detect a difference in proportion; however, it may also indicate that observed differences between racial and ethnic groups are likely associated with social factors and comorbidities. Our results are consistent with prior work demonstrating mixed findings (ie, lower rates of amyloid positivity among non-White individuals^44^ vs no racial and ethnic differences in amyloid PET positivity^27,28,29^). Our findings of lower odds and proportion among Asian participants may be difficult to interpret due to lower prevalence of clinical AD in this population and different social and cultural factors among Asian subgroups. Although rates of hypertension among Asian American individuals is lower in the US in general,^45,46^ rates of hypertension are higher among some Asian groups (eg, Vietnamese and Korean individuals)^45,47^ and hypertension may be underdiagnosed among Asian individuals compared to other groups.^48^

Association of family history of dementia with positive amyloid PET scan may be indicative of shared genetic features that were unmeasured in this study. A family history of AD or dementia in a first-degree relative has previously been associated with increased risk of development of AD and amyloid positivity.^49,50,51^ Genotyping of apolipoprotein E (*APOE)*, the most common gene associated with risk of sporadic AD, was not performed systematically in IDEAS and is an important limitation in this secondary data analysis. The *APOE* ε4 allele has a known association with amyloid positivity, but evidence suggests this risk may vary by racial and ethnic background.^52,53^ Among individuals in a study screening for an amyloid-lowering antibody, Deters et al^44^ demonstrated lower rates of amyloid PET positivity and continuous amyloid levels among African American individuals with *APOE4* and greater African ancestry on admixture analysis compared to *APOE4*-positive African American individuals with less African ancestry and APOE4-positive White individuals. Ethnic differences in single-nucleotide variants in the *APOE* region may also modify expression levels, thereby mitigating amyloid deposition and risk of AD in individuals with *E4* positivity and Asian or African ancestral backgrounds.^54^

### Strengths

Our study has several strengths. To our knowledge, it is the largest multisite study to date examining differences in amyloid PET positivity among Asian, Black, Hispanic, and White individuals with MCI or dementia. Previous studies examining racial and ethnic differences in amyloid PET positivity have largely focused on comparisons between smaller numbers of Black and White individuals, with greater representation of cognitively unimpaired older adults.^19,20,21,28,29,44,55^ Recently, increased attention has been called to disparities in screening criteria that have precluded non-White individuals from being included in AD research and clinical trials,^22^ which may partly explain lack of inclusion in prior research. Our study also incorporated multiple sociodemographic and comorbidity variables, allowing us to explore the association of these factors with rates of amyloid PET positivity.

### Limitations

Our study also has limitations. First, despite the numbers in the study, Asian, Black, and Hispanic individuals were underrepresented in IDEAS. Although pragmatic studies like IDEAS are expected to reflect more real-world settings than traditional clinical trials, pragmatic studies also reinforce existing structural and systemic issues that limit care for minoritized racial and ethnic groups, such as lack of access and referral to dementia specialists and cost of copay for PET (PET scans in IDEAS were covered by Medicare under coverage with evidence development and copays were not covered).^9,56,57,58^ Other areas of potential bias include selection bias and reliance on appropriate-use criteria to determine study eligibility. Our study did not collect comprehensive social and structural determinants of health data, such as early life quality of education, neighborhood deprivation, and experiences with discrimination among other variables, which are associated with dementia.^7,59,60,61,62^ We matched racial and ethnic groups based on available demographic characteristics, social factors, comorbidities, and family history but were unable to match on other factors and also unable to match a small percentage (less than 6% within each group) of Asian, Black, and Hispanic participants. Although we matched on MCI or dementia, there may be differences in progression to AD across groups. We used medical history of hypertension and diabetes as matching variables in our analysis but did not have data pertaining to treatment and control, which may vary between racial and ethnic groups as a result of disparities in access to health care, biases in treatment within the medical system, and other social determinants of health. As noted above, we also did not have genotyping of *APOE* for any of the participants. Additionally, the IDEAS database does not include a cognitively normal comparison group and longitudinal data are not yet available.

It is important to note that the categorization of individuals into racial and ethnic groups is based on social factors, not on biology or genetics. This categorization oversimplifies the tremendous heterogeneity (genetic and otherwise) that exists among members of these groups and our results should not be used to argue for some level of shared biology among members of a specific racial or ethnic group. Race itself is a sociocultural and political construct, and often serves as a proxy for social determinants of health, structural racism, and cultural and linguistic factors.

## Conclusions

In this large multisite practice-based study, we found lower odds of amyloid PET positivity in older Asian, Black, and Hispanic adults with MCI and dementia compared with non-Hispanic White individuals. These results have important implications for the diagnosis, treatment, and prevention of ADRD in groups that are at the highest risk of dementia. Future research should include racially and ethnically diverse cohorts that reflect the burden of ADRD in the population at large.

The recently launched New IDEAS^63^ study is focused on addressing these gaps by using multipronged recruitment and community engagement strategies to evaluate the clinical utility of amyloid PET in a more diverse cohort of patients with MCI or dementia, with a specific focus on recruiting Black and Hispanic Medicare beneficiaries.

## Notes

Supplement. eMethods eResults eReferences

## References

[1] Rajan KB, Weuve J, Barnes LL, Wilson RS, Evans DA. Prevalence and incidence of clinically diagnosed Alzheimer’s disease dementia from 1994 to 2012 in a population study. Alzheimers Dement. 2019;15(1):1-7. doi:10.1016/j.jalz.2018.07.216 30195482 PMC6531287

[2] Gurland BJ, Wilder DE, Lantigua R, . Rates of dementia in three ethnoracial group. Int J Geriatr Psychiatry. 1999;14(6):481-493. doi:10.1002/(SICI)1099-1166(199906)14:6<481::AID-GPS959>3.0.CO;2-5 10398359

[3] Chin AL, Negash S, Hamilton R. Diversity and disparity in dementia: the impact of ethnoracial differences in Alzheimer disease. Alzheimer Dis Assoc Disord. 2011;25(3):187-195. doi:10.1097/WAD.0b013e318211c6c9 21399486 PMC3396146

[4] Mayeda ER, Glymour MM, Quesenberry CP Jr, Whitmer RA. Heterogeneity in 14-year dementia incidence between Asian American subgroups. Alzheimer Dis Assoc Disord. 2017;31(3):181-186. doi:10.1097/WAD.0000000000000189 28406845 PMC5568954

[5] Kurian AK, Cardarelli KM. Racial and ethnic differences in cardiovascular disease risk factors: a systematic review. Ethn Dis. 2007;17(1):143-152. doi:10.13016/rsqw-ztls 17274224

[6] Tom SE, Phadke M, Hubbard RA, Crane PK, Stern Y, Larson EB. Association of demographic and early-life socioeconomic factors by birth cohort with dementia incidence among US adults born between 1893 and 1949. JAMA Netw Open. 2020;3(7):e2011094. doi:10.1001/jamanetworkopen.2020.11094 32716513 PMC8794045

[7] Coogan P, Schon K, Li S, Cozier Y, Bethea T, Rosenberg L. Experiences of racism and subjective cognitive function in African American women. Alzheimers Dement (Amst). 2020;12(1):e12067. doi:10.1002/dad2.12067 32782921 PMC7409101

[8] Lin PJ, Emerson J, Faul JD, . Racial and ethnic differences in knowledge about one’s dementia status. J Am Geriatr Soc. 2020;68(8):1763-1770. doi:10.1111/jgs.16442 32282058 PMC7552114

[9] Murchison CF, Kennedy RE, McConathy JE, Roberson ED. Racial differences in Alzheimer’s disease specialist encounters are associated with usage of molecular imaging and dementia medications: an enterprise-wide analysis using i2b2. J Alzheimers Dis. 2021;79(2):543-557. doi:10.3233/JAD-200796 33337364 PMC7902957

[10] Manly JJ. Critical issues in cultural neuropsychology: Profit from diversity. Neuropsychol Rev. 2008;18:179-183. doi:10.1007/s11065-008-9068-8 18814033 PMC2759971

[11] Gasquoine PG. Race-norming of neuropsychological tests. Neuropsychol Rev. 2009;19(2):250-262. doi:10.1007/s11065-009-9090-5 19294515

[12] Rabinovici GD. Controversy and progress in Alzheimer’s disease—FDA approval of aducanumab. N Engl J Med. 2021;385(9):771-774. doi:10.1056/NEJMp2111320 34320284

[13] Hyman BT, Phelps CH, Beach TG, . National Institute on Aging–Alzheimer’s Association guidelines for the neuropathologic assessment of Alzheimer’s disease. Alzheimers Dement. 2012;8(1):1-13. doi:10.1016/j.jalz.2011.10.007 22265587 PMC3266529

[14] Garrett SL, McDaniel D, Obideen M, . Racial disparity in cerebrospinal fluid amyloid and tau biomarkers and associated cutoffs for mild cognitive impairment. JAMA Netw Open. 2019;2(12):e1917363. doi:10.1001/jamanetworkopen.2019.17363 31834392 PMC6991300

[15] Howell JC, Watts KD, Parker MW, . Race modifies the relationship between cognition and Alzheimer’s disease cerebrospinal fluid biomarkers. Alzheimers Res Ther. 2017;9(1):88. doi:10.1186/s13195-017-0315-1 29096697 PMC5668981

[16] Walker KA, Power MC, Hoogeveen RC, . Midlife systemic inflammation, late-life white matter integrity, and cerebral small vessel disease: the Atherosclerosis Risk in Communities study. Stroke. 2017;48(12):3196-3202. doi:10.1161/STROKEAHA.117.018675 29101255 PMC5705320

[17] Manly JJ, Glymour MM. What the aducanumab approval reveals about Alzheimer disease research. JAMA Neurol. 2021;78(11):1305-1306. doi:10.1001/jamaneurol.2021.3404 34605885

[18] Gottesman RF, Schneider ALC, Zhou Y, . Association between midlife vascular risk factors and estimated brain amyloid deposition. JAMA. 2017;317(14):1443-1450. doi:10.1001/jama.2017.3090 28399252 PMC5921896

[19] Babulal GM, Quiroz YT, Albensi BC, ; International Society to Advance Alzheimer’s Research and Treatment, Alzheimer’s Association. Perspectives on ethnic and racial disparities in Alzheimer’s disease and related dementias: update and areas of immediate need. Alzheimers Dement. 2019;15(2):292-312. doi:10.1016/j.jalz.2018.09.009 30555031 PMC6368893

[20] Lee CM, Jacobs HIL, Marquié M, . 18F-Flortaucipir binding in choroid plexus: related to race and hippocampus signal. J Alzheimers Dis. 2018;62(4):1691-1702. doi:10.3233/JAD-170840 29614677 PMC5957532

[21] McDonough IM. Beta-amyloid and cortical thickness reveal racial disparities in preclinical Alzheimer’s disease. Neuroimage Clin. 2017;16:659-667. doi:10.1016/j.nicl.2017.09.014 29868439 PMC5984571

[22] Raman R, Quiroz YT, Langford O, . Disparities by race and ethnicity among adults recruited for a preclinical Alzheimer disease trial. JAMA Netw Open. 2021;4(7):e2114364. doi:10.1001/jamanetworkopen.2021.14364 34228129 PMC8261604

[23] Rabinovici GD, Gatsonis C, Apgar C, . Association of amyloid positron emission tomography with subsequent change in clinical management among Medicare beneficiaries with mild cognitive impairment or dementia. JAMA. 2019;321(13):1286-1294. doi:10.1001/jama.2019.2000 30938796 PMC6450276

[24] Noel-Storr AH, McCleery JM, Richard E, . Reporting standards for studies of diagnostic test accuracy in dementia: the STARDdem Initiative. Neurology. 2014;83(4):364-373. doi:10.1212/WNL.0000000000000621 24944261 PMC4115600

[25] Brown LD, Cai TT, DasGupta A. Interval estimation for a binomial proportion. Stat Sci. 2001;16(2):101-133. doi:10.1214/ss/1009213286

[26] Brookhart MA, Schneeweiss S, Rothman KJ, Glynn RJ, Avorn J, Stürmer T. Variable selection for propensity score models. Am J Epidemiol. 2006;163(12):1149-1156. doi:10.1093/aje/kwj149 16624967 PMC1513192

[27] Morris JC, Schindler SE, McCue LM, . Assessment of racial disparities in biomarkers for Alzheimer disease. JAMA Neurol. 2019;76(3):264-273. doi:10.1001/jamaneurol.2018.4249 30615028 PMC6439726

[28] Gottesman RF, Schneider ALC, Zhou Y, . The ARIC-PET amyloid imaging study: brain amyloid differences by age, race, sex, and *APOE*. Neurology. 2016;87(5):473-480. doi:10.1212/WNL.0000000000002914 27371485 PMC4970663

[29] Duara R, Loewenstein DA, Lizarraga G, . Effect of age, ethnicity, sex, cognitive status and *APOE* genotype on amyloid load and the threshold for amyloid positivity. Neuroimage Clin. 2019;22:101800. doi:10.1016/j.nicl.2019.101800 30991618 PMC6447735

[30] Dorans KS, Mills KT, Liu Y, He J. Trends in prevalence and control of hypertension according to the 2017 American College of Cardiology/American Heart Association (ACC/AHA) guideline. J Am Heart Assoc. 2018;7(11):e008888. doi:10.1161/JAHA.118.008888 29858369 PMC6015372

[31] Fei K, Rodriguez-Lopez JS, Ramos M, . Racial and ethnic subgroup disparities in hypertension prevalence, New York City health and nutrition examination survey, 2013-2014. Prev Chronic Dis. 2017;14(4):E33. doi:10.5888/pcd14.160478 28427484 PMC5420441

[32] Al Kibria GM. Racial/ethnic disparities in prevalence, treatment, and control of hypertension among US adults following application of the 2017 American College of Cardiology/American Heart Association guideline. Prev Med Rep. 2019;14:100850. doi:10.1016/j.pmedr.2019.100850 31061780 PMC6488531

[33] Justin Thomas S, Booth JN, Dai C, . Cumulative incidence of hypertension by 55 years of age in Blacks and Whites: the CARDIA study. J Am Heart Assoc. 2018;7(14). doi:10.1161/JAHA.117.007988 PMC6064834 29997132

[34] Chen C, Zissimopoulos JM. Racial and ethnic differences in trends in dementia prevalence and risk factors in the United States. Alzheimers Dement (N Y). 2018;4:510-520. doi:10.1016/j.trci.2018.08.009 30364652 PMC6197734

[35] Verhaaren BFJ, Vernooij MW, de Boer R, . High blood pressure and cerebral white matter lesion progression in the general population. Hypertension. 2013;61(6):1354-1359. doi:10.1161/HYPERTENSIONAHA.111.00430 23529163

[36] Firbank MJ, Wiseman RM, Burton EJ, Saxby BK, O’Brien JT, Ford GA. Brain atrophy and white matter hyperintensity change in older adults and relationship to blood pressure. brain atrophy, WMH change and blood pressure. J Neurol. 2007;254(6):713-721. doi:10.1007/s00415-006-0238-4 17446997

[37] Uiterwijk R, Staals J, Huijts M, de Leeuw PW, Kroon AA, van Oostenbrugge RJ. MRI progression of cerebral small vessel disease and cognitive decline in patients with hypertension. J Hypertens. 2017;35(6):1263-1270. doi:10.1097/HJH.0000000000001294 28169884

[38] Kitagawa K, Miwa K, Yagita Y, Okazaki S, Sakaguchi M, Mochizuki H. Association between carotid stenosis or lacunar infarction and incident dementia in patients with vascular risk factors. Eur J Neurol. 2015;22(1):187-192. doi:10.1111/ene.12553 25164480

[39] Thal DR, Grinberg LT, Attems J. Vascular dementia: different forms of vessel disorders contribute to the development of dementia in the elderly brain. Exp Gerontol. 2012;47(11):816-824. doi:10.1016/j.exger.2012.05.023 22705146 PMC3470831

[40] Takenoshita N, Shimizu S, Kanetaka H, . Classification of clinically diagnosed Alzheimer’s disease associated with diabetes based on amyloid and tau PET results. J Alzheimers Dis. 2019;71(1):261-271. doi:10.3233/JAD-190620 31356213

[41] Frison E, Proust-Lima C, Mangin JF, ; MEMENTO Cohort Study Group. Diabetes mellitus and cognition: pathway analysis in the MEMENTO cohort. Neurology. 2021;97(8):e836-e848. doi:10.1212/WNL.0000000000012440 34210821 PMC8397583

[42] Bailey ZD, Krieger N, Agénor M, Graves J, Linos N, Bassett MT. Structural racism and health inequities in the USA: evidence and interventions. Lancet. 2017;389(10077):1453-1463. doi:10.1016/S0140-6736(17)30569-X 28402827

[43] Letang SK, Lin SSH, Parmelee PA, McDonough IM. Ethnoracial disparities in cognition are associated with multiple socioeconomic status-stress pathways. Cogn Res Princ Implic. 2021;6(1):64. doi:10.1186/s41235-021-00329-7 34626254 PMC8502192

[44] Deters KD, Napolioni V, Sperling RA, . Amyloid PET imaging in self-identified non-Hispanic Black participants of the Anti-Amyloid in Asymptomatic Alzheimer’s Disease (A4) study. Neurology. 2021;96(11):e1491-e1500. doi:10.1212/WNL.0000000000011599 33568538 PMC8032379

[45] Fang J, Luncheon C, Patel A, . Self-reported prevalence of hypertension and antihypertensive medication use among Asian Americans: behavioral risk factor surveillance system 2013, 2015 and 2017. J Immigr Minor Health. 2021;23(1):26-34. doi:10.1007/s10903-020-01032-3 32451693 PMC10880142

[46] Singh GK, Daus GP, Allender M, . Social determinants of health in the United States: addressing major health inequality trends for the nation, 1935-2016. Int J MCH AIDS. 2017;6(2):139-164. doi:10.21106/ijma.236 29367890 PMC5777389

[47] Jung MY, Lee S, Thomas SB, Juon HS. Hypertension prevalence, treatment, and related behaviors among Asian Americans: an examination by method of measurement and disaggregated subgroups. J Racial Ethn Health Disparities. 2019;6(3):584-593. doi:10.1007/s40615-018-00557-6 30618006 PMC6500469

[48] Kim EJ, Kim T, Conigliaro J, Liebschutz JM, Paasche-Orlow MK, Hanchate AD. Racial and ethnic disparities in diagnosis of chronic medical conditions in the USA. J Gen Intern Med. 2018;33(7):1116-1123. doi:10.1007/s11606-018-4471-1 29736755 PMC6025658

[49] Sperling RA, Donohue MC, Raman R, ; A4 Study Team. Association of factors with elevated amyloid burden in clinically normal older individuals. JAMA Neurol. 2020;77(6):735-745. doi:10.1001/jamaneurol.2020.0387 32250387 PMC7136861

[50] Cannon-Albright LA, Foster NL, Schliep K, . Relative risk for Alzheimer disease based on complete family history. Neurology. 2019;92(15):e1745-e1753. doi:10.1212/WNL.0000000000007231 30867271 PMC6511086

[51] Honea RA, Vidoni ED, Swerdlow RH, Burns JM; Alzheimer’s Disease Neuroimaging Initiative. Maternal family history is associated with Alzheimer’s disease biomarkers. J Alzheimers Dis. 2012;31(3):659-668. doi:10.3233/JAD-2012-120676 22669011 PMC3608420

[52] Jack CR, Wiste HJ, Weigand SD, . Sex and *APOE* effects on memory performance, neurodegeneration, and B-amyloid across the adult lifespan. Alzheimer’s Dement. 2014;10(4S pt3):228-229. doi:10.1016/j.jalz.2014.04.325

[53] Deters K, Napolioni V, Greicius MD, Mormino BC. African ancestry moderates the effect of *APOE*4 on cognitive decline. Alzheimer’s Dement. 2018;14(7S pt19):1027-1028. doi:10.1016/j.jalz.2018.06.2804

[54] Choi KY, Lee JJ, Gunasekaran TI, . *APOE* promoter polymorphism-219T/G is an effect modifier of the influence of *APOE* ε4 on Alzheimer’s disease risk in a multiracial sample. J Clin Med. 2019;8(8):E1236. doi:10.3390/jcm8081236 31426376 PMC6723529

[55] Meeker KL, Wisch JK, Hudson D, . Socioeconomic status mediates racial differences seen using the AT(N) framework. Ann Neurol. 2021;89(2):254-265. doi:10.1002/ana.25948 33111990 PMC7903892

[56] Drabo EF, Barthold D, Joyce G, Ferido P, Chang Chui H, Zissimopoulos J. Longitudinal analysis of dementia diagnosis and specialty care among racially diverse Medicare beneficiaries. Alzheimers Dement. 2019;15(11):1402-1411. doi:10.1016/j.jalz.2019.07.005 31494079 PMC6874742

[57] Witte MM, Foster NL, Fleisher AS, . Clinical use of amyloid-positron emission tomography neuroimaging: practical and bioethical considerations. Alzheimers Dement (Amst). 2015;1(3):358-367. doi:10.1016/j.dadm.2015.06.006 27239516 PMC4878065

[58] Clark CM, DeCarli C, Mungas D, . Earlier onset of Alzheimer disease symptoms in latino individuals compared with Anglo individuals. Arch Neurol. 2005;62(5):774-778. doi:10.1001/archneur.62.5.774 15883265

[59] Lövdén M, Fratiglioni L, Glymour MM, Lindenberger U, Tucker-Drob EM. Education and cognitive functioning across the life span. Psychol Sci Public Interest. 2020;21(1):6-41. doi:10.1177/1529100620920576 32772803 PMC7425377

[60] Powell WR, Buckingham WR, Larson JL, . Association of neighborhood-level disadvantage with Alzheimer disease neuropathology. JAMA Netw Open. 2020;3(6):e207559. doi:10.1001/jamanetworkopen.2020.7559 32525547 PMC7290421

[61] Sisco S, Gross AL, Shih RA, . The role of early-life educational quality and literacy in explaining racial disparities in cognition in late life. J Gerontol B Psychol Sci Soc Sci. 2015;70(4):557-567. doi:10.1093/geronb/gbt133 24584038 PMC4462668

[62] Barnes LL, Lewis TT, Begeny CT, Yu L, Bennett DA, Wilson RS. Perceived discrimination and cognition in older African Americans. J Int Neuropsychol Soc. 2012;18(5):856-865. doi:10.1017/S1355617712000628 22595035 PMC3432700

[63] New IDEAS Study Team. New IDEAS: imaging dementia-evidence for amyloid scanning study. Posted June 11, 2022. https://clinicaltrials.gov/ct2/show/NCT04426539

