# Supplementary material for: Racial and Ethnic Differences in Amyloid PET Positivity in Individuals With Mild Cognitive Impairment or Dementia: A Secondary Analysis of the Imaging Dementia–Evidence for Amyloid Scanning (IDEAS) Cohort Study
Source: JAMA Neurol. 2022 Oct 3;79(11):1139–47. doi: 10.1001/jamaneurol.2022.3157 (PMC9531087; doi:10.1001/jamaneurol.2022.3157)
Supplement: Supplement. — eMethods eResults eReferences [file jamaneurol-e223157-s001.pdf]

Supplemental Online Content

Wilkins CH, Windon CC, Dilworth-Anderson P, et al. Racial and ethnic differences in amyloid PET positivity in individuals with mild cognitive impairment or dementia: a secondary analysis of the Imaging Dementia–Evidence for Amyloid Scanning (IDEAS) cohort study. *JAMA Neurol*. Published online October 3, 2022. doi:10.1001/jamaneurol.2022.3157

eMethods
eResults
eReferences

This supplemental material has been provided by the authors to give readers additional information about their work.

© 2022 Wilkins CH et al. *JAMA Neurology.*

eMethods

Inclusion and Exclusion Criteria in IDEAS

The inclusion criteria for the IDEAS study included: 1) 65 and older; 2) Medicare beneficiary with Medicare as primary insurance; 3) Diagnosis of MCI or dementia, according to DSM-IV and/or National Institutes of Aging-Alzheimer's Association criteria, verified by a dementia specialist within 24 months[1-3]; 4) Meets Appropriate use criteria (Cognitive complaint with objectively confirmed impairment; The etiologic cause of cognitive impairment is uncertain after a comprehensive evaluation by a dementia specialist, including general medical and neurological examination, mental status testing including standard measures of cognitive impairment, laboratory testing, and structural neuroimaging as below; Alzheimer's disease is a diagnostic consideration; Knowledge of amyloid PET status is expected to alter diagnosis and management.); 5) Head MRI and/or CT within 24 months prior to enrollment; 6) Clinical laboratory assessment (complete blood count [CBC], standard blood chemistry profile, thyroid stimulating hormone [TSH], vitamin B12) within the 12 months prior to enrollment; 7) Able to tolerate amyloid PET required by protocol, to be performed at a participating PET facility; 8) English or Spanish speaking (for the purposes of informed consent); 9) Willing and able to provide consent. Consent may be by proxy.

Exclusion criteria for IDEAS included: 1) Normal cognition or subjective complaints that are not verified by cognitive testing; 2) Knowledge of amyloid status, in the opinion of the referring dementia expert, may cause significant psychological harm or otherwise negatively impact the patient or family; 3) Amyloid status already known to patient or referring clinician based on prior amyloid imaging or cerebrospinal fluid analysis; 4) Current or previous enrollment in an anti-amyloid therapeutic trial; 5) Scan is being ordered solely based on a family history of dementia, presence of apolipoprotein E (APOE) 4, or in lieu of genotyping for suspected autosomal mutation carriers; 6) Scan being ordered for nonmedical purposes (e.g., legal, insurance coverage, or employment screening); 7) Cancer requiring active therapy (excluding non-

© 2022 Wilkins CH et al. *JAMA Neurology.*

melanoma skin cancer); 8) Hip/pelvic fracture within the 12 months prior to enrollment; 9) Body weight exceeds PET scanner weight limit; 10) Life expectancy less than 24 months based on medical co-morbidities; 11) Residence in a skilled nursing facility.

(https://cdn.jamanetwork.com/ama/content_public/journal/jama/937922/joi190021supp1_prod.pdf?Expires=1659472364&Signature=WjHQR89YuHwGKKecqwqX5cI8h~fF0O0cTUcmhm3T9KJYHweyQqPaxRrV0n66VJjVeTEJ-BvemHRvoCTh8qIz~bt4pgp4PSUVnF3nBKRHTx4ciXbimeaSBaWTngFGPiwEvIb5dSDiI4dHFfNwctlygtPnLAnDBiS6vKFw4si1Qo~3OSeQHv5z6honakNNYbTr9O1h3qYDjiYJpyT1wY3wU-b4xEIDEDf9kNubb9GQ3HKXy53VCYVGyD5St7gBNhnx0Ju5rbNxaIw7pePLRAwVLTOQFOKnJpGUZXPOzmrChDXAoSWwmqSpFfkZ6H8ZAUxjD2NysW15zvOUotKSKMmiOw__&Key-Pair-Id=APKAIE5G5CRDK6RD3PGA).

Race and Ethnicity Reporting in IDEAS

In IDEAS, race and ethnicity of participants was recorded by dementia specialists into a minimum of 1 race category (American Indian, Alaskan Native, Asian, Black or African American, Native Hawaiian or Pacific Islander, White, Not reported, Unknown) and a minimum of 1 ethnicity category (Hispanic or Latino, not Hispanic or Latino, not reported, unknown). Dementia specialists were not given instruction by the study protocol or registration form on how race and ethnicity were to be determined[4], therefore, we do not if or which participants were directly asked to self-identify race and ethnicity.

Amyloid PET Scan Interpretation and Communication of Results in IDEAS

In the IDEAS study, amyloid PET scans were interpreted by radiologists or nuclear medicine physicians at respective PET facilities that agreed to participate in the IDEAS study. Each radiologist or nuclear medicine physician provided a dichotomized interpretation of the amyloid PET scan (positive or negative). All radiologists and nuclear medicine physicians who participated in the interpretation of PET scans had completed vendor-provided online or in-person training courses specific to the amyloid imaging agent(s) used at his or her PET facility. Amyloid PET scan reads were then uploaded to the IDEAS database within 7 days of

© 2022 Wilkins CH et al. *JAMA Neurology.*

completing the PET scan read. Additional information regarding PET scan interpretation and report submission can be found in the original protocol

(https://cdn.jamanetwork.com/ama/content_public/journal/jama/937922/joi190021supp1_prod.pdf?Expires=1659472364&Signature=WjHQR89YuHwGKKecqwqX5cI8h~fF0O0cTUcmhm3T9KJYHweyQqPaxRrV0n66VJjVeTEJ-BvemHRvoCTh8qIz~bt4pgp4PSUVnF3nBKRHTx4ciXbimeaSBaWTngFGPiwEvIb5dSDiI4dHFfNwctIygtPnLAnDBiS6vKFw4si1Qo~3OSeQHv5z6honakNNYbTr9O1h3qYDjiYJpyT1wY3wU-b4xEIDEDf9kNubb9GQ3HKXy53VCYVGyD5St7gBNhnx0Ju5rbNxaIw7pePLRAwVLTOQFOKnJpGUZXPOzmrChDXAoSWwmqSpFfkZ6H8ZAUxjD2NysW15zvOUotKSKMmiOw__&Key-Pair-Id=APKAIE5G5CRDK6RD3PGA).

Optimal Matching Strategy

To compare the amyloid PET positivity rate between racial and ethnic minority groups and White participants, optimal 1:1 matching (Black to White, Hispanic to White, Asian to White; all 3 White groups unique) to obtain minimal distances between matches using network flow optimization methods[5-7] created balanced groups with respect to their baseline characteristics. Variables used for matching were recorded in case report forms at IDEAS study entry and included age (matching within ± 3 years), sex, highest level of education attained, living arrangement (with whom do you reside; coded into alone/not alone for purposes of analyses), history of hypertension, history of diabetes, family history of dementia, and level of impairment (MCI or dementia).

© 2022 Wilkins CH et al. *JAMA Neurology.*

eResults

Because higher percentages of Black, Hispanic/Latinx and Asian patients had vascular disease risk factors (e.g., diabetes) and were more likely to have dementia (vs MCI) relative to White patients, we examined whether there were differences in ADRD subtype across racial and ethnic categories. Past medical history of stroke, TIA, and CVD were similar across racial and ethnic groups (Supplemental Table 5). Pre-PET diagnosis of vascular cognitive impairment was similar across the groups, however, pre-PET diagnosis of mixed AD pathology was higher among Black (15.75%) and Hispanic (13.15%) participants compared to White (10.78%) and Asian (10.28%) (Supplemental Table 6). No additional imaging data is available to examine cerebrovascular disease.

Supplemental Table 1 – Recoding Separate IDEAS Race and Ethnicity Questions

**IDEAS race and ethnicity questions**		**Race and Ethnicity used for the analysis**
**Race (Select all that apply)**	**Hispanic origin**	
White only	Not Hispanic or Latino, Not reported, or Unknown	White
Black or African American only	Not Hispanic or Latino, Not reported, or Unknown	Black
Asian only	Not Hispanic or Latino, Not reported, or Unknown	Asian
Only one of<br>• American Indian<br>• Alaska Native<br>• Native Hawaiian or Pacific Islander	Not Hispanic or Latino, Not reported, or Unknown	Indigenous

© 2022 Wilkins CH et al. *JAMA Neurology.*

Only one of:<br><br>• White<br>• Black or African American<br>• Asian<br>• American Indian<br>• Alaska Native<br>• Native Hawaiian or Pacific Islander<br>• Not reported<br>• Unknown	Hispanic or Latino	Hispanic
More than one of:<br><br>• White<br>• Black or African American<br>• Asian<br>• American Indian<br>• Alaska Native<br>• Native Hawaiian or Pacific Islander	Any ethnicity	Multiracial
Not reported or Unknown	Not Hispanic or Latino, Not reported, or Unknown	Not reported or Unknown

© 2022 Wilkins CH et al. *JAMA Neurology.*

Supplemental Table 2 - Results From Optimal 1:1 Matching

	Matched participants		Matched participants		Matched participants	
**Variable**	**Asian (n=313)**	**White (n=313)**	**Black (n=615)**	**White (n=615)**	**Hispanic (n=780)**	**White (n=780)**
Age, median (range), years	76 (65-91)	76 (65-91)	75 (65-95)	75 (65-93)	76 (65-93)	76 (65-92)
Sex, No. (%)						
Male	150 (47.9)	150 (47.9)	226 (36.7)	226 (36.7)	303 (38.8)	303 (38.8)
Female	163 (52.1)	163 (52.1)	389 (63.3)	389 (63.3)	477 (61.2)	477 (61.2)
Education, No. (%)						
Less than high school	40 (12.8)	40 (12.8)	101 (16.4)	101 (16.4)	279 (35.8)	279 (35.8)
High school (including equivalency)	62 (19.8)	62 (19.8)	215 (35.0)	215 (35.0)	201 (25.8)	201 (25.8)
Some college or associate degree	40 (12.8)	40 (12.8)	129 (21.0)	129 (21.0)	149 (19.1)	149 (19.1)
Bachelor’s degree	98 (31.3)	98 (31.3)	88 (14.3)	88 (14.3)	74 (9.5)	74 (9.5)
Master’s degree	29 (9.3)	29 (9.3)	55 (8.9)	55 (8.9)	42 (5.4)	42 (5.4)
Doctorate	44 (14.1)	44 (14.1)	27 (4.4)	27 (4.4)	35 (4.5)	35 (4.5)
Living arrangement, No. (%)						
Patient lives alone	35 (11.2)	35 (11.2)	146 (23.7)	146 (23.7)	115 (14.7)	115 (14.7)
Patient lives with at least one other person	278 (88.8)	278 (88.8)	469 (76.3)	469 (76.3)	665 (85.3)	665 (85.3)
History of hypertension, No. (%)						
No	169 (54.0)	169 (54.0)	193 (31.4)	193 (31.4)	351 (45.0)	351 (45.0)

© 2022 Wilkins CH et al. *JAMA Neurology.*

Yes	144 (46.0)	144 (46.0)	422 (68.6)	422 (68.6)	429 (55.0)	429 (55.0)
History of diabetes, No. (%)						
No	240 (76.7)	240 (76.7)	448 (72.8)	448 (72.8)	595 (76.3)	595 (76.3)
Yes	73 (23.3)	73 (23.3)	167 (27.2)	167 (27.2)	185 (23.7)	185 (23.7)
Family history of dementia, No. (%)						
No	273 (87.2)	273 (87.2)	516 (83.9)	516 (83.9)	647 (82.9)	647 (82.9)
Yes	40 (12.8)	40 (12.8)	99 (16.1)	99 (16.1)	133 (17.1)	133 (17.1)
Impairment level, No. (%)						
MCI	168 (53.7)	168 (53.7)	302 (49.1)	302 (49.1)	356 (45.6)	356 (45.6)
Dementia	145 (46.3)	145 (46.3)	313 (50.9)	313 (50.9)	424 (54.4)	424 (54.4)

MCI = mild cognitive impairment

© 2022 Wilkins CH et al. *JAMA Neurology.*

Supplemental Table 3 – Race and ethnicity of participants that did not have an amyloid PET scan performed

	Eligible (N=21,630)	Amyloid PET scan not performed (N=3,337)
**Race or Ethnicity**	**N**	**N (%)**
Asian	394	73 (18.5)
Black	806	169 (21.0)
Hispanic	1,119	290 (25.9)
White	17,801	2,452 (13.8)
Other	41	9 (22.0)
Multiracial	37	6 (16.2)
Not reported or Unknown	1,432	338 (23.6)

Note: Percentages are out of the number of participants in each race or ethnicity that are eligible.

© 2022 Wilkins CH et al. *JAMA Neurology.*

Supplemental Table 4 – Race/Ethnicity of participants that did not have an amyloid PET scan performed by highest level of education

Education	Race and Ethnicity							
	Asian	Black	Hispanic	White	Other	Multiracial	Not reported or Unknown	Total
Less than high school	9 (12.3)	21 (12.4)	102 (35.2)	130 (5.3)	0 (0.0)	1 (16.7)	49 (14.5)	312 (9.3)
High school (including equivalency)	10 (13.7)	56 (33.1)	86 (29.7)	679 (27.7)	2 (22.2)	2 (33.3)	88 (26.0)	923 (27.7)
Some college or associate degree	13 (17.8)	44 (26.0)	37 (12.8)	614 (25.0)	3 (33.3)	2 (33.3)	62 (18.3)	775 (23.2)
Bachelor's degree	15 (20.5)	18 (10.7)	20 (6.9)	415 (16.9)	2 (22.2)	0 (0.0)	50 (14.8)	520 (15.6)
Master's degree	5 (6.8)	9 (5.3)	4 (1.4)	185 (7.5)	0 (0.0)	0 (0.0)	24 (7.1)	227 (6.8)
Doctorate	10 (13.7)	4 (2.4)	6 (2.1)	111 (4.5)	1 (11.1)	0 (0.0)	12 (3.6)	144 (4.3)
Missing	11 (15.1)	17 (10.1)	35 (12.1)	318 (13.0)	1 (11.1)	1 (16.7)	53 (15.7)	436 (13.1)
Total	73 (100.0)	169 (100.0)	290 (100.0)	2,452 (100.0)	9 (100.0)	6 (100.0)	338 (100.0)	3,337 (100.0)

Note: Percentages are column percentages

Supplemental Table 5 – Select past/current medical history variables by race and ethnicity

Variable	Race and Ethnicity			
	White (n=15,322)	Hispanic/Latino (n=829)	Black/African American (n=635)	Asian (n=321)
History of stroke or TIA, No. (%)	1,606 (10.5)	75 (9.0)	67 (10.6)	31 (9.7)
History of CVD without stroke, No. (%)	794 (5.2)	56 (6.8)	35 (5.5)	16 (5.0)
Total	2,400 (15.7)	131 (15.8)	102 (16.1)	47 (14.7)

Supplemental Table 6 Primary pre-PET differential diagnosis by race and ethnicity

Pre-PET primary differential diagnosis	Race and Ethnicity			
	Asian	Black/African American	Hispanic/Latino	White
Vascular cognitive impairment	19	37	45	839
	5.92%	5.83%	5.43%	5.48%
AD, mixed pathology	33	100	109	1,652
	10.28%	15.75%	13.15%	10.78%

eReferences

1. American Psychiatric Association. (2000). *Diagnostic and statistical manual of mental disorders* (4th ed., text rev.).
2. McKhann GM, Knopman DS, Chertkow H, et al. The diagnosis of dementia due to Alzheimer's disease: recommendations from the National Institute on Aging-Alzheimer's Association workgroups on diagnostic guidelines for Alzheimer's disease. *Alzheimers Dement*. 2011;7(3):263-269. doi:10.1016/j.jalz.2011.03.005
3. Albert MS, DeKosky ST, Dickson D, et al. The diagnosis of mild cognitive impairment due to Alzheimer's disease: recommendations from the National Institute on Aging-Alzheimer's Association workgroups on diagnostic guidelines for Alzheimer's disease. *Alzheimers Dement*. 2011;7(3):270-279. doi:10.1016/j.jalz.2011.03.008
4. Rabinovici GD, Gatsonis C, Apgar C, et al. Association of Amyloid Positron Emission Tomography With Subsequent Change in Clinical Management Among Medicare Beneficiaries With Mild Cognitive Impairment or Dementia. *JAMA*. 2019;321(13):1286-1294. doi:10.1001/jama.2019.2000
5. Rosenbaum P. Optimal Matching for Observational Studies. *Journal of the American Statistical Association*. 1989; 84(408): 1024-1032.
6. Carre, B. (1979), Graphs and Networks, New York: Oxford University Press
7. Ford, L., and Fulkerson, D. (1962), Flows in Networks, Princeton, NJ: Princeton University Press
